# Genome Sequence of Erythromelalgia-Related Poxvirus Identifies it as an Ectromelia Virus Strain

**DOI:** 10.1371/journal.pone.0034604

**Published:** 2012-04-27

**Authors:** Jorge D. Mendez-Rios, Craig A. Martens, Daniel P. Bruno, Stephen F. Porcella, Zhi-Ming Zheng, Bernard Moss

**Affiliations:** 1 Laboratory of Viral Diseases, National Institute of Allergy and Infectious Diseases, National Institutes of Health, Bethesda, Maryland, United States of America; 2 Department of Cell Biology and Molecular Genetics, University of Maryland, College Park, Maryland, United States of America; 3 Research Technologies Section, Rocky Mountain Laboratories, National Institute of Allergy and Infectious Diseases, National Institutes of Health, Hamilton, Montana, United States of America; 4 HIV and AIDS Malignancy Branch, National Cancer Institute, Bethesda, Maryland, United States of America; Univ. of Texas HSC at San Antonio, United States of America

## Abstract

Erythromelagia is a condition characterized by attacks of burning pain and inflammation in the extremeties. An epidemic form of this syndrome occurs in secondary students in rural China and a virus referred to as erythromelalgia-associated poxvirus (ERPV) was reported to have been recovered from throat swabs in 1987. Studies performed at the time suggested that ERPV belongs to the orthopoxvirus genus and has similarities with ectromelia virus, the causative agent of mousepox. We have determined the complete genome sequence of ERPV and demonstrated that it has 99.8% identity to the Naval strain of ectromelia virus and a slighly lower identity to the Moscow strain. Small DNA deletions in the Naval genome that are absent from ERPV may suggest that the sequenced strain of Naval was not the immediate progenitor of ERPV.

## Introduction

Erythromelalgia is a condition in which there are attacks of burning pain and inflammation in the extremities. Primary hereditary erythromelalgia is a rare disease caused by a mutation in a voltage-gated sodium channel subunit [1], [2]. Non-hereditary erythromelalgia has an incidence of approximately 1.3 per 100,000, occurring most frequently in women with a median age of 61, and can have multiple causes [3]. In rural China, outbreaks of erythromelalgia have occurred during the winter and spring at several year intervals among secondary school students [4]–[8]. In a 1987 epidemic, many students reported pharyngitis prior to the symptoms of erythromelalgia suggesting a possible connection with a respiratory tract infection [9]. Virus isolates from throat swabs of six individuals in three locations suffering from erythromelalgia were characterized [4], [10]. In five cases the virus was isolated directly in cell culture and in another was first passaged in mice [11]. In addition, the sera from patients with epidemic erythromelalgia were reported to have a higher incidence of ERPV antibody (39.2%) compared to non-symptomatic local students (11.8%) and sera of controls from the United States (11.9%) [12]. Electron microscopic examinations indicated that the isolated virus belongs to the poxvirus family [13]. Further analysis of the biological, serological and pathogenic properties suggested that erythromelalgia-related poxvirus (ERPV) is a member of the orthopoxvirus genus [11]. A restriction enzyme profile of the ERPV DNA resembled but was distinguishable from a Chinese strain of ectromelia virus (ECTV), the causative agent of mousepox [14]. The susceptibility of mice to ERPV and the formation of A-type inclusion bodies in the cytoplasm were also consistent with ECTV. However, there were apparent differences between the Chinese strain of ECTV and ERPV with regard to pock morphology on the chicken chorioallantoic membrane, pathogenicity for rabbits, and the ability of ERPV to be neutralized by anti-vaccinia virus and anti-ECTV sera from rabbits but not vice-versa [11]. Moreover, ECTV is not known to cause disease in humans. In contrast, human infections are known to occur with other orthopoxviruses including variola virus (smallpox), cowpox virus, monkeypox virus and vaccinia virus [15].

Poxviruses are large double-stranded DNA viruses [16]. The availability of Next Generation sequencing technologies allowed us to sequence and analyze the genome of ERPV. We compared the ERPV genome sequence to that of the complete genome sequences of the Moscow (ECTV-Mos) [17] and Naval (ECTV-Nav) [18] (www.poxvirus.org) strains of ECTV and determined that it closely resembled the latter with only minor differences.

## Results

### Sequence of the ERPV Genome

**Figure 1:**
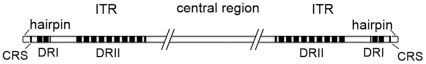
Representation of an orthopoxvirus genome. A typical genome consisting of a single dsDNA molecule with a concatemer resolution sequence (CRS), sets of direct repeats (DRI and DRII) and a hairpin loop on each inverted terminal repeat (ITR) is shown.

**Figure 2:**
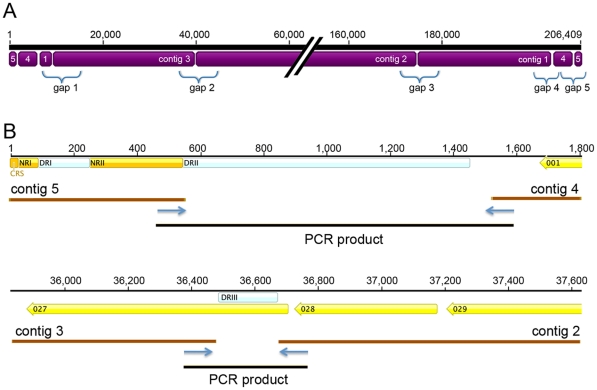
Assembly of contigs and gap closure. (A) Five contigs were assembled de novo using 159,077 sequence reads generated by pyrosequencing, providing an estimated coverage of 60X with 5 gaps. (B) The gaps were filled by PCR and Sanger sequencing. Blue arrows indicate positions of primers used for PCR. Gaps 2 and 5 contained direct repeats (DRs) necessitating synthesis and sequencing of additional internal PCR fragments. DRI contains a 69 bp sequence repeated 2.3X; DRII contained an 85 bp sequence repeated 10.4X; and DRIII contained a 25 bp sequence repeated 7.0X. The non-repetitive I (NRI) and NRII sequences flank DRI. ORFs are indicated by numbered yellow arrows.

The genomes of orthopoxviruses are approximately 200,000 base pairs (bp) with two long inverted terminal repetitions (ITRs); within each ITR there are usually a few open reading frames (ORFs), sets of short direct repeats (DRs), a unique concatemer resolution sequence (CRS), and a terminal covalently closed hairpin loop (Fig. 1). ERPV was obtained from the American Type Culture Collection, clonally purified and amplified in monkey kidney BS-C-1 cells in a laboratory that had no previous exposure to ECTV. ERPV was partially purified from cell lysates by sedimentation through a sucrose cushion and the DNA was isolated and prepared for 454 pyrosequencing. Of 159,077 sequence reads, 54,227 were identified as viral by filtering out host cellular sequences. De novo assembly generated five contigs with read depth or coverage at 63X and these contigs were provisionally placed in sequential order using ECTV-Mos as a reference template, which was the closest genome match in the NCBI database (Fig. 2A). The contig order was confirmed and gaps between contigs were filled by polymerase chain reaction (PCR) and Sanger sequencing, providing a complete, de novo genome sequence of ERPV except for the hairpin ends. Three sets of DRs were found of which two (DRI and DRII) were present in the ITR separated by 316 bp and one (DRIII) in the unique region within the continuous open reading frame designated F1L in the VACV genome (Fig. 2B). DRII and DRIII were present in gaps between contigs 5 and 4 and 3 and 2, respectively. DRI contained a 69 bp sequence repeated 2.3X; DRII contained an 85 bp sequence repeated 10.4X; and DRIII contained a 25 bp sequence repeated 7.0X. The length of the ERPV genome was determined to be 206,409 bp from the start of the highly conserved 19 bp CRS [19] near one end to the same sequence at the other end omitting the short hairpin sequences. The ITRs were each 7,022 bp and the unique central region was 192,365 bp.

### Comparison of ERPV with ECTV Strains

**Figure 3:**
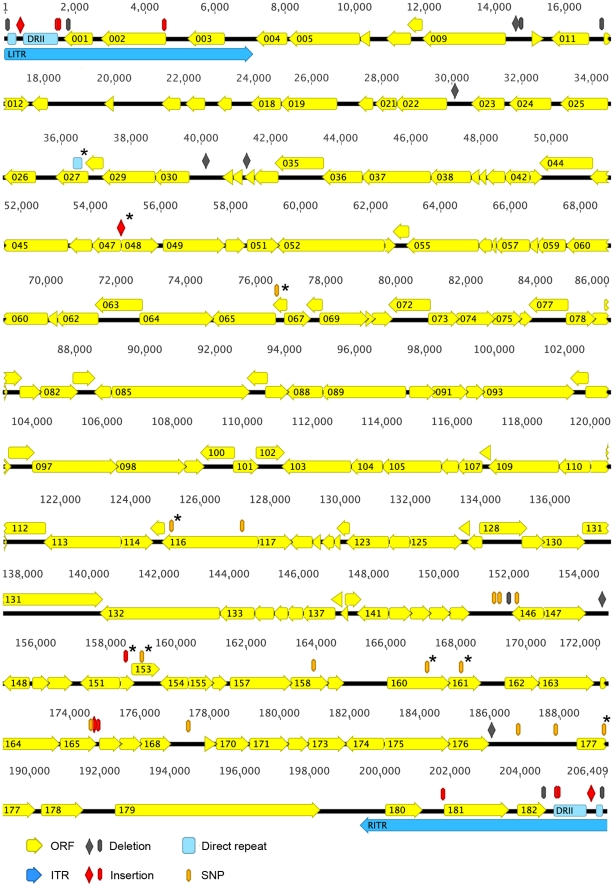
Genome map of ERPV and comparison to ECTV-Naval. Left (LITR) and right (RITR) inverted terminal repeats are indicated by deep blue arrows. ORFs are in yellow and numbered from left to right with the direction of transcription indicated by the arrow. Direct repeats (DRs) are indicated in light blue. Single nucleotide polymorphisms (SNPs) are gold; insertions and deletions are indicated in red and purple, respectively, with single nt and larger changes by a thin oval and a diamond, respectively. Asterisks signify mutations that affect the predicted amino acid sequence.

Although the ECTV-Mos was the only essentially complete ECTV genome sequence in the NCBI database [17], the annotated genome sequence of ECTV-Nav was posted in www.poxvirus.org [18]. The two ECTV genome sequences were only missing the hairpin loops and adjacent nucleotides (nt). The ERPV genome was annotated using GATU (Genome Annotation Transfer Utility) [20] with the ECTV-Nav genome as the reference. The genomes of ERPV, ECTV-Nav and ECTV-Mos were compared and the general features summarized in Table 1. We noted that the publically available ECTV-Mos sequence started 10 nucleotides (nt) downstream of the CRS, and that the ECTV-Nav sequence included part of the hairpin loop and the CRS. For comparison, we estimated genome sizes starting from the first nt of the left CRS to a nt before the right CRS, and we corrected for the 10 nt missing on both ends of ECTV-Mos. ERPV had a 99.8% nt sequence identity with ECTV-Nav and 98.4% identity with ECTV-Mos. The small differences in the overall genome lengths were mainly attributable to the ITRs. Each nt difference affecting the length of an ERPV ORF relative to an ECTV-Nav ORF was checked by PCR and Sanger sequencing and manually corrected. All 183 ORFs of ECTV-Nav had orthologs in ERPV and of these 173 were identical in sequence. Further comparison of ERPV and ECTV-Nav revealed nine mutations predicted to affect protein sequence in the ten non-identical ORFs that were confirmed by PCR of ERPV DNA and resequencing. These differences represented single nt polymorphisms, a short truncation or extension, and a reduction in the number of short repeats (Table 2). However, the 10 ERPV ORFs that differed from ECTV-Nav were identical to ORFs in ECTV-Mos. An ORF map of the ERPV genome illustrating differences from ECTV-Nav in the coding and non-coding sequences is shown in Fig. 3. For reference, the annotated ORFs of ERPV, ECTV-Nav, ECTV-Mos and CPXV are compared in Table S1.

**Table 1:** Comparison of genomes of ERPV and ECTV-Nav and ECTV-Mos.

Virus	ERPV	ECTV-Nav	ECTV-Mos
Genome length (bp)	206,409	207,516^a^	209,829^a^
ITR length (bp)	7,022	7,325	9,442
% GC	33.2	33.1	33.0
Annotated ORFs	183	183^b^	178^b^
Identical ORFs	–	173	145
% identity to ERPV	–	99.8	98.4

a Genome sizes are from the first nt of the left CRS to the nt before the right CRS.

b The ORF number includes the homolog of O3, which was not originally annotated in ECTV-Nav or ECTV-Mos.

**Table 2:** Summary of ERPV mutations predicted to affect proteins relative to ECTV-Nav.

ORF^a^	Size^b^		VACV-COP^c^	Description of mutations
	ERPV	ECTV-Nav		
027	281	425	F1L	ERPV contains fewer DRs
048	331	341	E5R	ERPV has an “AT” insertion
066	111	111	G3L	V66A change in ERPV
116	891	891	A10L	R236G, V881A changes in ERPV
152	125	99	A45R	Single “A” missing in Nav
153	240	240	A46R	S67P change in ERPV
160	563	563	A55R	N358D change in ERPV
161	281	281	A56R	Y139D change in ERPV
177	559	559	A55R	M241V change in ERPV

a ORF numbers correspond to those in Figure 3 for ERPV and ECTV-Nav.

b Size refers to number of amino acids in predicted protein.

c Corresponding ORF designations in Copenhagen strain of VACV.

The ERPV genome contained an additional 33 ORFs with homology to longer CPXV ORFs that had not been annotated previously in ECTV genomes (Table 3). Of these 17 had identical sequences in ERPV, ECTV-Mos and ECTV-Nav; an additional 10 were identical in ECTV-Nav; and 2 were identical in ECTV-Mos. However, because of their fragmentation, none of the 33 ORFs are likely to be functional in either ECTV or ERPV.

**Table 3:** Unassigned ORFs with homology to CPXV proteins.

ORF	CPXV homolog	ERPV (aa)	CPXV (aa)	Start	End	Alignment length	aa Identity	E-value
1^a^	CPXV002 CPXV228	77	66	1648	1415	56	66%	4.00E-14
2^c^	CPXV008 CPXV223	57	673	4963	4790	52	94%	4.00E-26
3^b^	CPXV220	152	580	6649	6191	152	89%	5.00E-74
4	CPXV220	76	580	6945	6715	62	77%	2.00E-24
5	CPXV013	62	524	10828	10640	58	72%	3.00E-17
6^b^	CPXV025	87	669	18699	18436	87	91%	2.00E-42
7	CPXV025	68	669	19080	18874	62	95%	4.00E-29
8^b^	CPXV025	77	669	19365	19132	76	87%	7.00E-37
9^b^	CPXV027	204	633	21188	20574	193	80%	4.00E-85
10^b^	CPXV028	72	186	21432	21214	72	92%	4.00E-36
11	CPXV028	75	186	21656	21429	51	94%	2.00E-25
12^b^	CPXV033	176	317	24217	23687	177	88%	7.00E-91
13^b^	CPXV036	75	232	27017	26790	73	95%	2.00E-38
14^b^	CPXV036	62	232	27337	27149	61	97%	5.00E-32
15^a^	CPXV040	143	221	30273	29842	126	94%	2.00E-69
16	CPXV040	57	221	30430	30259	33	88%	3.00E-08
17	CPXV046	73	150	35540	35761	70	83%	4.00E-30
18^b^	CPXV052	62	324	40301	40113	59	86%	9.00E-29
19	CPXV173	91	264	151204	151479	79	96%	7.00E-41
20	CPXV177	82	161	154283	154531	74	85%	8.00E-32
21	CPXV195	72	198	168448	168666	72	99%	5.00E-40
22	CPXV195	74	198	168748	168972	54	98%	8.00E-28
23	CPXV204	107	502	177396	177719	97	91%	9.00E-52
24	CPXV213	158	801	185953	186429	165	83%	9.00E-57
25	CPXV213	82	801	186413	186661	76	78%	4.00E-33
26^b^	CPXV213	433	801	186670	187971	421	95%	0
27	CPXV213	61	801	188157	188342	64	69%	2.00E-20
28	CPXV220	63	580	198542	198733	61	87%	2.00E-25
29	CPXV220	127	580	198909	199292	100	86%	7.00E-47
30	CPXV220	76	580	199465	199695	62	77%	2.00E-24
31^b^	CPXV220	152	580	199761	200219	152	89%	5.00E-74
32^c^	CPXV008 CPXV223	57	673	201447	201620	52	94%	4.00E-26
33^a^	CPXV002 CPXV228	77	66	204762	204995	56	66%	4.00E-14

a Similar in ECTV-Mos.

b Similar in ECTV-Nav.

c Missing from ECTV-Mos. Others are identical in the three genomes.

Abbreviation: aa, amino acids.

### Comparison of ERPV and Other Orthopoxviruses

**Figure 4:**
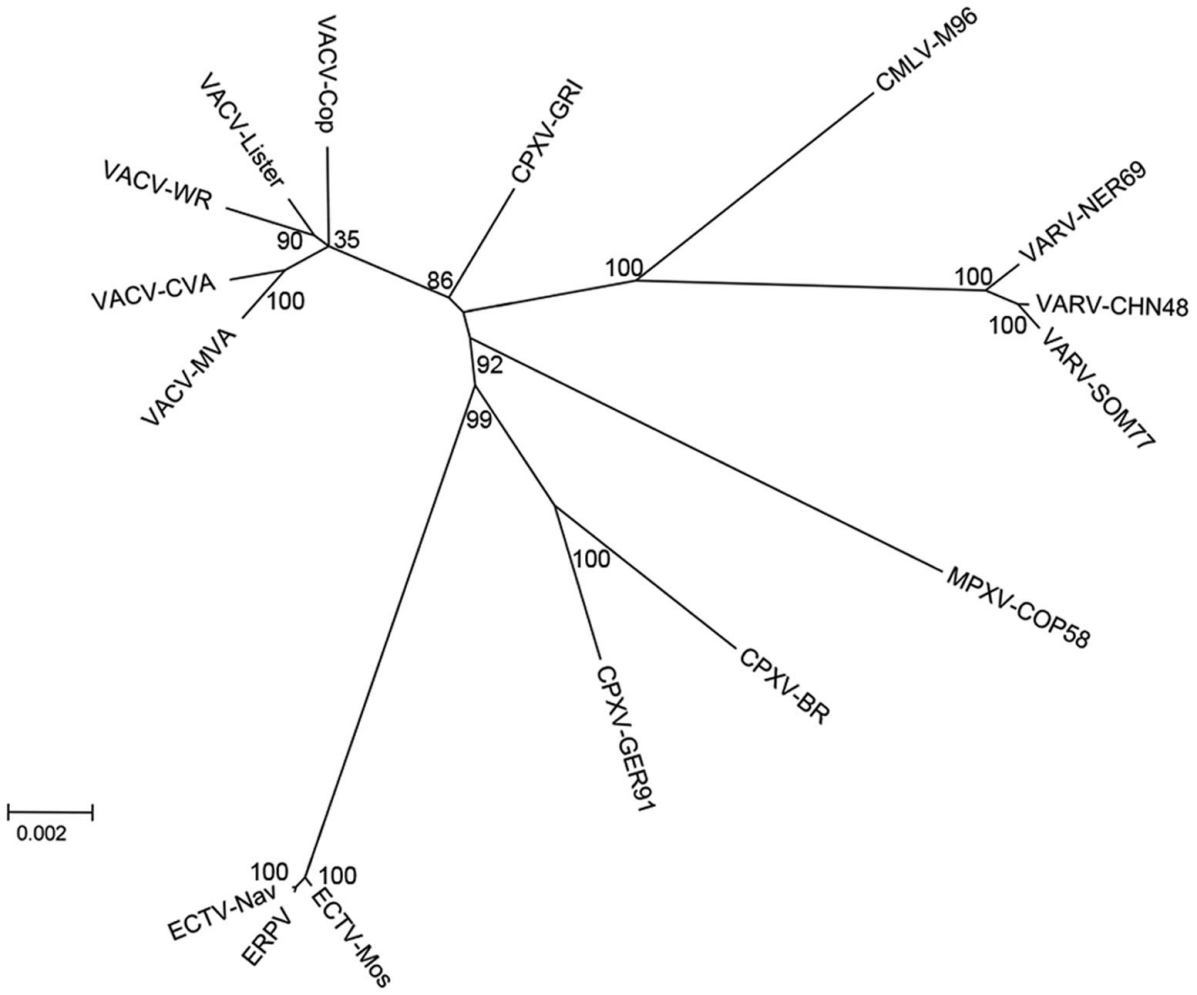
Phylogenetic tree of orthopoxviruses including ERPV. Concatenated sequences of 96 ORFs conserved in each viral genome were used to perform the analysis. ERPV branches from the same node as ECTV.

We constructed a phylogenetic tree using a catenation of 96 orthologous genes in order to compare ERPV with other orthopoxviruses. As expected, ERPV was closest to ECTV-Nav and ECTV-Mos (Fig. 4). The separation of ECTV from other orthopoxvirus genera is consistent with other analyses [17], [21], [22].

## Discussion

The complete genome sequence of ERPV, except for the terminal hairpin, was obtained by 454 pyrosequencing supplemented by Sanger sequencing of PCR fragments to span gaps between contigs, analyze regions with direct repeats and ITR junctions, and confirm or correct differences from orthologous ECTV genes. The nt sequence was remarkably similar to that of ECTV, particularly ECTV-Nav with which it is 99.8% identical. The main structural differences consisted of small deletions and variation in the number of repetitive elements within the ITRs and no differences that would be expected to have biological consequences were found. All 183 previously annotated ORFs of ECTV-Nav were represented in ERPV and of these 173 were identical in nt sequence and the 10 others had only small differences and were identical to ORFs of ECTV-Mos. It may be useful to reanalyze the ECTV-Nav sequences of these 10 ORFs to confirm whether there are true differences. The presence of a deletion in the ITR and small deletions within ORFs of ECTV-Nav relative to ERPV and ECTV-Mos, could suggest that the ECTV-Nav isolate used for sequencing was not the immediate progenitor of ERPV. Although some biological differences between ERPV and ECTV were previously reported [11], the ECTV was a Chinese isolate and its similarity to the ECTV-Nav was not determined.

The first ECTV isolate was the Hampstead strain, discovered in a laboratory mouse colony in London [23]. ECTV has been enzootic in the breeding stocks of mice in Europe, China and Japan [24]. ECTV outbreaks have been discovered in mouse colonies in many parts of the world including the United States [14] and there is one report of ECTV recovered from wild mice in Europe [25]. ECTV-Nav was first isolated from an outbreak at the Naval Medical Research Institute in Bethesda, MD and the source was identified as commercial mouse serum [26]. Other laboratory outbreaks of ECTV were traced to mouse sera from the United States and China [27], [28].

The identification of ERPV as a strain of ECTV is perplexing, given its reported isolation from the pharynx of students in rural China [11] and the absence of other reports of ECTV infection of humans. Because 5 of the 6 original virus isolations occurred in culture using two different cell lines with newborn bovine serum in the medium, the virus was considered unlikely to be a laboratory contaminant [11]. In addition, the statistically higher detection by immunofluorescence of antibody to ERPV A-type inclusions in the sera of patients with epidemic erythromelalgia as compared to that of non-diseased local students provided supportive evidence for the origin of ERPV [12]. Nevertheless, the latter finding should be interpreted with caution because of widespread smallpox vaccination with vaccinia virus at the time. Although vaccinia virus does not form A-type inclusions, antibodies to the homologous A-type inclusion proteins of ECTV and vaccinia virus are cross-reactive [29] and antibody to this protein is present in sera of smallpox vaccinees [30]. Thus, the conclusion of the present study is that ERPV is a strain of ECTV; additional studies would be needed to confirm that replication of ERPV or any ECTV strain can occur in humans and establish whether there is a true association of ERPV with epidemic erythromelalgia in China.

## Materials and Methods

### Cells and Virus Growth

ERPV was received from the American Type Culture Collection (VR-1431) and clonally purified by three successive plaque isolations on BS-C-1 cells (ATCC, CCL-26) and propagated in minimal Essential Medium with Earl’s balanced salts (Quality Biological, Gaithersburg, MD) supplemented with 2 mM L-Gln and 10% fetal bovine serum. All experiments were carried out in a laboratory with no known ECTV contact.

### Virus Purification and DNA Extraction

Infected BS-C-1 cells from five T-150 flasks were harvested and the cell pellet was re-suspended in 10 ml of 1 mM Tris-HCl, pH 9.0 and lysed with 40 strokes of a tight pestle Dounce homognizer. Nuclei and cell debris were removed by centrifugation at 300×g for 5 min at 5°C. The supernatant was then sonicated three times and the viral suspension was overlaid on a 17 ml 36% sucrose cushion and centrifuged at 32,900×g as described [31]. The virus pellet was suspended in 1 mM Tris-HCl pH 7.8 and incubated for 4 h at 37°C in a solution containing 10% SDS, 60% sucrose and 10 mg/ml of proteinase K [31]. DNA was extracted with phenol:chloroform:isoamyl alchohol (25∶24∶1) and then with isopropanol and precipitated with 100% ethanol containing 0.3M sodium chloride [32]. Viral DNA was confirmed by HindIII digestion and gel electrophoresis.

### Library Preparation and Pyrosequencing

Samples were quantified using absorbance at 260 nm (A260) and a Picogreen assay (Life Technologies, Grand Island, NY). Separate libraries were constructed using *Rapid Library Preparation Method Manual (October 2009) GS FLX Titanium Series* (Roche, Branford, CT) and *Paired End Library Preparation Method Manual – 3kb Span (October 2009) GS FLX Titanium Series*. Each library was processed using *emPCR Method Manual – Lib-L MV (October 2009)* in separate emulsion reactions with the fragment library being combined with like samples. The paired-end sample was loaded on a single lane and the fragment sample was loaded in two lanes of an 8-region 454 GS FLX Titanium sequencing run.

### Assembly and Completion of the Genome Sequence

Paired-end and fragment reads were assembled using GS Assembler v.2.5 (Roche/454 Life Sciences), using standard assembly parameters. *De novo* assembly resulted in five contigs with an estimated length of 200,971 nt. The five contigs of ERPV were provisionally ordered by comparison with the genome sequence of ECTV-Mos (Accession NC_004105), which had the highest score on a BLAST search of the NCBI genome database, using the bioinformatics tools Mummer [33] and Geneious pro 5.5 (Drummond A. J., Ashton B., Buxton S., Cheung M., Cooper A., Duran C., Field M., Heled J., Kearse M., Markowitz S., Moir R., Stones-Havas S., Sturrock S., Thierer T., Wilson A. 2011, Available from http://www.geneious.com/). After identification of the ITR, a reverse complementary version of it was generated and concatenated into to the genome draft. Primers were designed based on the physical location and gaps, followed by PCR and dual strand Sanger sequencing. The 5-contig genome was then assembled using the additional Sanger sequence reads. All single nt polymorphisms located within coding regions were verified or manually corrected by PCR amplification with flanking primers and +/− strand Sanger sequencing. The genome sequence including part of the hairpin loop contained in one of the contigs was deposited in GenBank (Submission No. 1506279; accession No. JQ410350).

### Determination of Sequence Differences between ERPV and ECTV Strains

Prior to comparing ERPV and ECTV genomes, repetitive sequences were masked using the Phobos Software plugin for Geneious Pro 5.5 software and each genome was truncated by removing the right ITR. The genomes were aligned using ClustalW2 [34], [35] at the EMBL-EBI website and compared pairwise. The ends of the alignments were hand edited using Geneious Pro 5.5 Software for optimization purpose. All mutations on coding and non-coding regions were identified.

### Genome Annotation and ORF Comparison

The Genome Annotation Transfer Utility (GATU) [20] was used for annotation of ERPV based on the ECTV-Naval sequence. The criteria for annotation included a cut-off of at least 180 nt, 60% nt similarity score threshold, and less than 50% of overlap to other ORFs. The transferred annotations were back-compared to ECTV-Nav and ECTV-Mos genomes. Every mutation affecting an ORF relative to ECTV-Nav was confirmed by PCR and re-sequencing. ORFs not previously annotated in ECTV-Nav were designated “unassigned ORFs”. All ORFs were translated and compared to the predicted protein sequence from ECTV-Nav (www.poxvirus.org), ECTV-Mos (Accession NC_004105), CPXV-BR (Accession NC_003663) and VACV-COP (Accession M35027) using an in-home tool for comparative genomics called MyOrfeome (Mendez-Rios JD, MyOrfeome, Internet: http://myorfeome.sourceforge.net). All sequences were obtained from www.poxvirus.org. Protein alignments were visually evaluated and used to curate and correct for alternative start sites.

### Whole-genome Alignment and Phylogeny

Complete proteomes of representative poxviruses were downloaded from www.poxvirus.org. Using the FASTA description, all proteins were imported and indexed on a MySQL database. By using the index, we identified 96 ORFs that were present as a single copy on each of the selected taxa. We then extracted and aligned all orthologs. After clustalw2 alignments of the amino acid sequences, all 96 datasets were concatenated for phylogeny analysis. An unrooted tree was generated by Maximum Likelyhood (ML)+ JTT method, with 1,000 boot-strap replications using MEGA Software [36].

## Supporting Information

Table S1 **ERPV genome annotations and comparison to ECTV-Nav, ECTV-Mos and CPXV.**
(DOC)
