# Supplementary material for: Genome Sequence of Erythromelalgia-Related Poxvirus Identifies it as an Ectromelia Virus Strain
Source: PLoS One. 2012 Apr 27;7(4):e34604. doi: 10.1371/journal.pone.0034604 (PMC3338725; doi:10.1371/journal.pone.0034604)
Supplement: Table S1 — ERPV genome annotations and comparison to ECTV-Nav, ECTV-Mos and CPXV. (DOC) [file pone.0034604.s001.doc]

Table S1. ERPV genome annotations and comparison to ECTV-Nav, ECTV-Mos and CPXV

| ERPV ORFs | VACV-COP | Predicted protein size (aa) | | | | BLAST against Naval | | Coordinates | |
| --- | --- | --- | --- | --- | --- | --- | --- | --- | --- |
|  |  | ERPV | ECTV-Nav | ECTV- Mos | CPXV | Alignment length | e-value | Start | End |
| ERPV_001 | C23L | 247 | 247 | 247 | 246 | 247 | 4.00E-145 | 2478 | 1735 |
| ERPV_002 | C21L; C20L; C19L | 587 | 587 | 587 | 619 | 587 | 0 | 4571 | 2808 |
| ERPV_003 | - | 320 | 320 | 320 | 320 | 320 | 0 | 6243 | 5281 |
| ERPV_004 | - | 273 | 273 | 273 | - | 273 | 5.00E-161 | 8043 | 7222 |
| ERPV_005 | - | 650 | 650 | 650 | 658 | 650 | 0 | 10114 | 8162 |
| ERPV_006 | - | 86 | 86 | 75 | - | 86 | 2.00E-48 | 10406 | 10146 |
| ERPV_007 | - | 202 | 202 | 202 | 202 | 202 | 5.00E-117 | 11568 | 10960 |
| ERPV_008 | - | 111 | 111 | 111 | 110 | 111 | 4.00E-62 | 11900 | 11565 |
| ERPV_009 | - | 763 | 763 | 763 | 764 | 763 | 0 | 14282 | 11991 |
| ERPV_010 | C11R | 83 | 83 | 83 | 139 | 83 | 2.00E-46 | 15107 | 15358 |
| ERPV_011 | C10L | 331 | 331 | 331 | 331 | 331 | 0 | 16667 | 15672 |
| ERPV_012 | - | 242 | 242 | 241 | 242 | 242 | 5.00E-142 | 17177 | 17905 |
| ERPV_013 | - | 126 | 126 | 138 | 126 | 126 | 1.00E-73 | 18436 | 18056 |
| ERPV_014 | - | 63 | 63 | 59 | - | 63 | 1.00E-27 | 20314 | 20123 |
| ERPV_015 | C7L | 150 | 150 | 150 | 150 | 150 | 2.00E-85 | 22233 | 21781 |
| ERPV_016 | C6L | 155 | 155 | 155 | 155 | 155 | 7.00E-89 | 22928 | 22461 |
| ERPV_017 | C5L | 106 | 106 | - | 125 | 106 | 6.00E-58 | 23684 | 23364 |
| ERPV_018 | C3L | 262 | 262 | 262 | 263 | 262 | 1.00E-153 | 25102 | 24314 |
| ERPV_019 | C2L | 512 | 512 | 512 | 512 | 512 | 0 | 26696 | 25158 |
| ERPV_020 | N1L | 117 | 117 | 117 | 117 | 117 | 3.00E-64 | 27736 | 27383 |
| ERPV_021 | N2L | 177 | 177 | 177 | 177 | 177 | 1.00E-104 | 28398 | 27865 |
| ERPV_022 | M1L | 472 | 472 | 472 | 473 | 472 | 0 | 29849 | 28431 |
| ERPV_023 | K1L | 285 | 285 | 285 | 284 | 285 | 1.00E-167 | 31481 | 30624 |
| ERPV_024 | K2L | 373 | 373 | 373 | 373 | 373 | 0 | 32827 | 31706 |
| ERPV_025 | K4L | 424 | 424 | 372 | 424 | 424 | 0 | 34442 | 33168 |
| ERPV_026 | K5L | 276 | 276 | 277 | 276 | 276 | 3.00E-167 | 35294 | 34464 |
| ERPV_027 | F1L | 281 | 425 | 456 | 251 | 261 | 7.00E-151 | 36788 | 35943 |
| ERPV_028 | F2L | 147 | 147 | 147 | 147 | 147 | 2.00E-85 | 37231 | 36788 |
| ERPV_029 | F3L | 481 | 481 | 482 | 480 | 481 | 0 | 38712 | 37267 |
| ERPV_030 | F4L | 319 | 319 | 319 | 333 | 319 | 0 | 39682 | 38723 |
| ERPV_031 | F6L | 72 | 72 | 74 | 71 | 72 | 3.00E-38 | 40928 | 40710 |
| ERPV_032 | F7L | 80 | 80 | 82 | 81 | 80 | 2.00E-44 | 41185 | 40943 |
| ERPV_033 | F8L | 65 | 65 | 65 | 65 | 65 | 4.00E-35 | 41528 | 41331 |
| ERPV_034 | F9L | 212 | 212 | 212 | 212 | 212 | 3.00E-125 | 42224 | 41586 |
| ERPV_035 | F10L | 439 | 439 | 439 | 439 | 439 | 0 | 43530 | 42211 |
| ERPV_036 | F11L | 354 | 354 | 354 | 354 | 354 | 0 | 44617 | 43553 |
| ERPV_037 | F12L | 634 | 634 | 634 | 634 | 634 | 0 | 46580 | 44676 |
| ERPV_038 | F13L | 372 | 372 | 372 | 372 | 372 | 0 | 47731 | 46613 |
| ERPV_039 | F14L | 71 | 71 | 71 | 73 | 71 | 2.00E-36 | 47964 | 47749 |
| ERPV_040 | F14.5L | 49 | 49 | 49 | 49 | 49 | 2.00E-22 | 48157 | 48008 |
| ERPV_041 | F15L | 158 | 158 | 158 | 158 | 158 | 2.00E-91 | 48705 | 48229 |
| ERPV_042 | F16L | 231 | 231 | 231 | 231 | 231 | 7.00E-136 | 49407 | 48712 |
| ERPV_043 | F17R | 101 | 101 | 101 | 101 | 101 | 5.00E-58 | 49469 | 49774 |
| ERPV_044 | E1L | 479 | 479 | 479 | 479 | 479 | 0 | 51210 | 49771 |
| ERPV_045 | E2L | 737 | 737 | 737 | 737 | 737 | 0 | 53420 | 51207 |
| ERPV_046 | E3L | 190 | 190 | 190 | 190 | 190 | 3.00E-111 | 54098 | 53526 |
| ERPV_047 | E4L | 259 | 259 | 259 | 261 | 259 | 1.00E-154 | 54933 | 54154 |
| ERPV_048 | E5R | 331 | 341 | 331 | 319 | 331 | 0 | 55014 | 56009 |
| ERPV_049 | E6R | 567 | 567 | 567 | 567 | 567 | 0 | 56206 | 57909 |
| ERPV_050 | E7R | 165 | 165 | 165 | 165 | 165 | 2.00E-98 | 57990 | 58487 |
| ERPV_051 | E8R | 273 | 273 | 273 | 273 | 273 | 2.00E-161 | 58615 | 59436 |
| ERPV_052 | E9L | 1006 | 1006 | 1006 | 1005 | 1006 | 0 | 62463 | 59443 |
| ERPV_053 | E10R | 95 | 95 | 95 | 95 | 95 | 2.00E-54 | 62495 | 62782 |
| ERPV_054 | E11L | 129 | 129 | 129 | 129 | 129 | 7.00E-73 | 63166 | 62777 |
| ERPV_055 | O1L | 666 | 666 | 666 | 666 | 666 | 0 | 65153 | 63153 |
| ERPV_056 | O2L | 108 | 108 | 108 | 108 | 108 | 3.00E-61 | 65523 | 65197 |
| ERPV_56.5 | O3 | 35 | 35 | 35 | 35 | 35 | 2.00E-16 | 65654 | 65547 |
| ERPV_057 | I1L | 312 | 312 | 312 | 312 | 312 | 0 | 66605 | 65667 |
| ERPV_058 | I2L | 73 | 73 | 73 | 73 | 73 | 6.00E-38 | 66833 | 66612 |
| ERPV_059 | I3L | 269 | 269 | 269 | 268 | 269 | 2.00E-157 | 67643 | 66834 |
| ERPV_060 | I4L | 771 | 771 | 771 | 771 | 771 | 0 | 70041 | 67726 |
| ERPV_061 | I5L | 79 | 79 | 79 | 79 | 79 | 4.00E-41 | 70307 | 70068 |
| ERPV_062 | I6L | 382 | 382 | 382 | 382 | 382 | 0 | 71474 | 70326 |
| ERPV_063 | I7L | 423 | 423 | 423 | 423 | 423 | 0 | 72738 | 71467 |
| ERPV_064 | I8R | 676 | 676 | 676 | 676 | 676 | 0 | 72744 | 74774 |
| ERPV_065 | G1L | 591 | 591 | 591 | 591 | 591 | 0 | 76552 | 74777 |
| ERPV_066 | G3L | 111 | 111 | 111 | 111 | 111 | 2.00E-61 | 76884 | 76549 |
| ERPV_067 | G2R | 220 | 220 | 220 | 220 | 220 | 2.00E-128 | 76878 | 77540 |
| ERPV_068 | G4L | 124 | 124 | 124 | 124 | 124 | 6.00E-71 | 77884 | 77510 |
| ERPV_069 | G5R | 434 | 434 | 434 | 434 | 434 | 0 | 77887 | 79191 |
| ERPV_070 | G5.5R | 63 | 63 | 63 | 63 | 63 | 6.00E-33 | 79199 | 79390 |
| ERPV_071 | G6R | 165 | 165 | 165 | 167 | 165 | 3.00E-97 | 79392 | 79889 |
| ERPV_072 | G7L | 371 | 371 | 371 | 371 | 371 | 0 | 80969 | 79854 |
| ERPV_073 | G8R | 260 | 260 | 260 | 260 | 260 | 1.00E-154 | 81000 | 81782 |
| ERPV_074 | G9R | 340 | 340 | 340 | 340 | 340 | 0 | 81802 | 82824 |
| ERPV_075 | L1R | 250 | 250 | 250 | 250 | 250 | 3.00E-150 | 82825 | 83577 |
| ERPV_076 | L2R | 87 | 87 | 87 | 88 | 87 | 1.00E-45 | 83609 | 83872 |
| ERPV_077 | L3L | 346 | 346 | 346 | 350 | 346 | 0 | 84902 | 83862 |
| ERPV_078 | L4R | 251 | 251 | 251 | 251 | 251 | 2.00E-146 | 84927 | 85682 |
| ERPV_079 | L5R | 128 | 128 | 128 | 128 | 128 | 5.00E-73 | 85692 | 86078 |
| ERPV_080 | J1R | 153 | 153 | 153 | 152 | 153 | 2.00E-87 | 86035 | 86496 |
| ERPV_081 | J2R | 177 | 177 | 177 | 177 | 177 | 2.00E-105 | 86512 | 87045 |
| ERPV_082 | J3R | 333 | 333 | 333 | 333 | 333 | 0 | 87110 | 88111 |
| ERPV_083 | J4R | 185 | 185 | 185 | 185 | 185 | 7.00E-106 | 88026 | 88583 |
| ERPV_084 | J5L | 133 | 133 | 133 | 133 | 133 | 6.00E-75 | 89049 | 88648 |
| ERPV_085 | J6R | 1286 | 1286 | 1286 | 1286 | 1286 | 0 | 89155 | 93015 |
| ERPV_086 | H1L | 171 | 171 | 171 | 171 | 171 | 1.00E-100 | 93527 | 93012 |
| ERPV_087 | H2R | 189 | 189 | 189 | 189 | 189 | 2.00E-113 | 93541 | 94110 |
| ERPV_088 | H3L | 324 | 324 | 324 | 325 | 324 | 0 | 95087 | 94113 |
| ERPV_089 | H4L | 794 | 794 | 794 | 795 | 794 | 0 | 97472 | 95088 |
| ERPV_090 | H5R | 212 | 212 | 212 | 206 | 212 | 7.00E-121 | 97658 | 98296 |
| ERPV_091 | H6R | 314 | 314 | 314 | 314 | 314 | 0 | 98297 | 99241 |
| ERPV_092 | H7R | 146 | 146 | 146 | 146 | 146 | 2.00E-84 | 99278 | 99718 |
| ERPV_093 | D1R | 843 | 843 | 843 | 844 | 843 | 0 | 99760 | 102291 |
| ERPV_094 | D2L | 146 | 146 | 146 | 146 | 146 | 2.00E-81 | 102690 | 102250 |
| ERPV_095 | D3R | 237 | 237 | 237 | 237 | 237 | 1.00E-140 | 102683 | 103396 |
| ERPV_096 | D4R | 218 | 218 | 218 | 218 | 218 | 2.00E-130 | 103396 | 104052 |
| ERPV_097 | D5R | 785 | 785 | 785 | 785 | 785 | 0 | 104084 | 106441 |
| ERPV_098 | D6R | 637 | 637 | 637 | 637 | 637 | 0 | 106482 | 108395 |
| ERPV_099 | D7R | 161 | 161 | 161 | 161 | 161 | 1.00E-93 | 108422 | 108907 |
| ERPV_100 | D8L | 304 | 304 | 304 | 304 | 304 | 0 | 109784 | 108870 |
| ERPV_101 | D9R | 213 | 213 | 213 | 213 | 213 | 3.00E-124 | 109826 | 110467 |
| ERPV_102 | D10R | 250 | 250 | 250 | 248 | 250 | 2.33E-156 | 110464 | 111216 |
| ERPV_103 | D11L | 631 | 631 | 631 | 631 | 631 | 0 | 113108 | 111213 |
| ERPV_104 | D12L | 287 | 287 | 287 | 287 | 287 | 9.00E-170 | 114005 | 113142 |
| ERPV_105 | D13L | 551 | 551 | 551 | 551 | 551 | 0 | 115691 | 114036 |
| ERPV_106 | A1L | 150 | 150 | 150 | 150 | 150 | 3.00E-86 | 116167 | 115715 |
| ERPV_107 | A2L | 224 | 224 | 224 | 224 | 224 | 1.00E-131 | 116862 | 116188 |
| ERPV_108 | A2.5L | 76 | 76 | 76 | 76 | 76 | 6.00E-42 | 117089 | 116859 |
| ERPV_109 | A3L | 644 | 644 | 644 | 644 | 644 | 0 | 119038 | 117104 |
| ERPV_110 | A4L | 281 | 281 | 281 | 295 | 281 | 5.00E-165 | 119936 | 119091 |
| ERPV_111 | A5R | 164 | 164 | 164 | 164 | 164 | 4.00E-94 | 119974 | 120468 |
| ERPV_112 | A6L | 372 | 372 | 372 | 372 | 372 | 0 | 121583 | 120465 |
| ERPV_113 | A7L | 710 | 710 | 710 | 710 | 710 | 0 | 123739 | 121607 |
| ERPV_114 | A8R | 288 | 288 | 288 | 288 | 288 | 1.00E-171 | 123793 | 124659 |
| ERPV_115 | A9L | 108 | 108 | 110 | 121 | 108 | 1.00E-60 | 124982 | 124656 |
| ERPV_116 | A10L | 891 | 891 | 891 | 894 | 891 | 0 | 127658 | 124983 |
| ERPV_117 | A11R | 318 | 318 | 318 | 318 | 318 | 0 | 127673 | 128629 |
| ERPV_118 | A12L | 191 | 191 | 191 | 190 | 191 | 2.00E-111 | 129206 | 128631 |
| ERPV_119 | A13L | 66 | 66 | 66 | 70 | 66 | 2.00E-34 | 129430 | 129230 |
| ERPV_120 | A14L | 90 | 90 | 90 | 90 | 90 | 1.00E-48 | 129811 | 129539 |
| ERPV_121 | A14.5L | 53 | 53 | 53 | 53 | 53 | 7.00E-26 | 129989 | 129828 |
| ERPV_122 | A15L | 94 | 94 | 94 | 94 | 94 | 2.00E-51 | 130263 | 129979 |
| ERPV_123 | A16L | 377 | 377 | 377 | 377 | 377 | 0 | 131380 | 130247 |
| ERPV_124 | A17L | 202 | 202 | 202 | 202 | 202 | 5.00E-117 | 131991 | 131383 |
| ERPV_125 | A18R | 493 | 493 | 493 | 492 | 493 | 0 | 132006 | 133487 |
| ERPV_126 | A19L | 77 | 77 | 77 | 77 | 77 | 1.00E-40 | 133701 | 133468 |
| ERPV_127 | A21L | 118 | 118 | 118 | 117 | 118 | 8.00E-66 | 134058 | 133702 |
| ERPV_128 | A20R | 426 | 426 | 426 | 426 | 426 | 0 | 134057 | 135337 |
| ERPV_129 | A22R | 187 | 187 | 187 | 187 | 187 | 1.00E-109 | 135267 | 135830 |
| ERPV_130 | A23R | 382 | 382 | 382 | 382 | 382 | 0 | 135850 | 136998 |
| ERPV_131 | A24R | 1164 | 1164 | 1164 | 1164 | 1164 | 0 | 136995 | 140489 |
| ERPV_132 | A26L | 1113 | 1113 | 1113 | 1284 | 1113 | 0 | 143823 | 140482 |
| ERPV_133 | - | 317 | 317 | 110 | 192 | 317 | 0 | 144822 | 143869 |
| ERPV_134 | A26L | 183 | 183 | - | 260 | 183 | 3.00E-108 | 145380 | 144829 |
| ERPV_135 | A27L | 110 | 110 | 110 | 110 | 110 | 3.00E-62 | 145764 | 145432 |
| ERPV_136 | A28L | 146 | 146 | 146 | 146 | 146 | 8.00E-85 | 146205 | 145765 |
| ERPV_137 | A29L | 305 | 305 | 305 | 305 | 305 | 0 | 147123 | 146206 |
| ERPV_138 | A30L | 77 | 77 | 77 | 76 | 77 | 8.00E-41 | 147319 | 147086 |
| ERPV_139 | A30.5L | 42 | 42 | 42 | 42 | 42 | 9.00E-20 | 147480 | 147352 |
| ERPV_140 | A31R | 126 | 126 | 126 | 140 | 126 | 5.00E-71 | 147479 | 147859 |
| ERPV_141 | A32L | 269 | 269 | 269 | 311 | 269 | 6.00E-161 | 148638 | 147829 |
| ERPV_142 | A33R | 185 | 185 | 185 | 187 | 185 | 5.00E-110 | 148756 | 149313 |
| ERPV_143 | A34R | 168 | 168 | 168 | 168 | 168 | 1.00E-99 | 149337 | 149843 |
| ERPV_144 | A35R | 176 | 176 | 176 | 176 | 176 | 5.00E-101 | 149886 | 150416 |
| ERPV_145 | A36R | 160 | 160 | 160 | 224 | 160 | 2.00E-92 | 150484 | 150966 |
| ERPV_146 | A38L | 277 | 277 | 277 | 277 | 277 | 2.00E-161 | 153081 | 152248 |
| ERPV_147 | A39R | 399 | 399 | 399 | 409 | 399 | 0 | 153096 | 154295 |
| ERPV_148 | A41L | 223 | 223 | 223 | 218 | 223 | 2.00E-132 | 155584 | 154913 |
| ERPV_149 | A42R | 134 | 134 | 134 | 133 | 134 | 2.00E-77 | 155746 | 156150 |
| ERPV_150 | A43R | 211 | 211 | 211 | 194 | 211 | 1.00E-121 | 156190 | 156825 |
| ERPV_151 | A44L | 346 | 346 | 346 | 345 | 346 | 0 | 158167 | 157127 |
| ERPV_152 | A45R | 125 | 99 | 125 | 125 | 95 | 6.00E-51 | 158215 | 158592 |
| ERPV_153 | A46R | 240 | 240 | 240 | 242 | 240 | 3.00E-141 | 158582 | 159304 |
| ERPV_154 | A47L | 244 | 244 | 244 | 244 | 244 | 2.00E-142 | 160125 | 159391 |
| ERPV_155 | A48R | 227 | 204 | 227 | 227 | 204 | 1.00E-121 | 160155 | 160838 |
| ERPV_156 | A49R | 116 | 116 | - | 162 | 116 | 8.00E-63 | 160888 | 161238 |
| ERPV_157 | A50R | 554 | 554 | 554 | 554 | 554 | 0 | 161411 | 163075 |
| ERPV_158 | A51R | 334 | 334 | 334 | 334 | 334 | 0 | 163128 | 164132 |
| ERPV_159 | A52R | 127 | 127 | - | 190 | 127 | 2.00E-70 | 164203 | 164586 |
| ERPV_160 | A55R | 563 | 563 | 563 | 563 | 563 | 0 | 165903 | 167594 |
| ERPV_161 | A56R | 281 | 281 | 281 | 297 | 281 | 7.00E-160 | 167644 | 168489 |
| ERPV_162 | B1R | 299 | 299 | 299 | 299 | 299 | 0 | 169257 | 170156 |
| ERPV_163 | B2R | 503 | 503 | 503 | 505 | 503 | 0 | 170223 | 171734 |
| ERPV_164 | B4R | 564 | 564 | 564 | 558 | 564 | 0 | 171971 | 173665 |
| ERPV_165 | B5R | 317 | 317 | 317 | 317 | 317 | 0 | 173752 | 174705 |
| ERPV_166 | B6R | 177 | 177 | 177 | 179 | 177 | 2.00E-102 | 174871 | 175404 |
| ERPV_167 | B7R | 181 | 181 | 181 | 181 | 181 | 2.00E-104 | 175443 | 175988 |
| ERPV_168 | B8R | 266 | 266 | 266 | 266 | 266 | 2.00E-159 | 176043 | 176843 |
| ERPV_169 | B11R | 87 | 87 | 87 | 90 | 87 | 2.00E-44 | 177875 | 178138 |
| ERPV_170 | B12R | 286 | 286 | 286 | 285 | 286 | 4.00E-172 | 178204 | 179064 |
| ERPV_171 | B14R | 344 | 344 | 344 | 341 | 344 | 0 | 179156 | 180190 |
| ERPV_172 | B15R | 164 | 164 | 164 | 149 | 164 | 1.00E-97 | 180269 | 180763 |
| ERPV_173 | B16R | 328 | 328 | 328 | 326 | 328 | 0 | 180850 | 181836 |
| ERPV_174 | B17L | 340 | 340 | 340 | 340 | 340 | 0 | 182913 | 181891 |
| ERPV_175 | B18R | 594 | 594 | 594 | 574 | 594 | 0 | 183003 | 184787 |
| ERPV_176 | B19R | 358 | 358 | 358 | 366 | 358 | 0 | 184848 | 185924 |
| ERPV_177 | A55R | 559 | 559 | 559 | 557 | 559 | 0 | 188508 | 190187 |
| ERPV_178 | C12L | 370 | 370 | 370 | 372 | 370 | 0 | 190446 | 191558 |
| ERPV_179 | COP_273 | 1924 | 1924 | 1924 | 1919 | 1924 | 0 | 192549 | 198323 |
| ERPV_180 | - | 320 | 320 | 320 | 320 | 320 | 0 | 200285 | 201247 |
| ERPV_181 | B25R; B26R; B27R | 587 | 587 | 587 | 619 | 587 | 0 | 201957 | 203720 |
| ERPV_182 | B29R | 247 | 247 | 247 | 246 | 247 | 4.00E-145 | 204050 | 204793 |
